# Quality of Life in Patients with an Implantable Cardioverter Defibrillator: A Systematic Review

**DOI:** 10.3389/fcvm.2015.00034

**Published:** 2015-11-03

**Authors:** Juliane Tomzik, Katharina C. Koltermann, Markus Zabel, Stefan N. Willich, Thomas Reinhold

**Affiliations:** ^1^Institute for Social Medicine, Epidemiology and Health Economics, Charité – Universitätsmedizin Berlin, Berlin, Germany; ^2^Department of Cardiology and Pneumology, University of Göttingen, Göttingen, Germany

**Keywords:** implantable defibrillator, quality of life, life style, anxiety, depression

## Abstract

Despite the indisputable mortality advantages of implantable cardioverter defibrillators (ICDs), no consensus exists regarding their impact on quality of life (QoL). This systematic review investigates differences in QoL between patients with ICDs and controls. We systematically searched the MEDLINE, EMBASE, Cochrane, Web of Science, and PsychINFO databases. Articles were included if they were published after the year 2000 and reported on original studies with a control group. Five randomized controlled trials with a total of 5,138 patients and 10 observational studies with a total of 1,513 patients met the inclusion criteria. Nine studies found comparable QoL for ICD recipients and patients in the control groups, three studies found an increased QoL for ICD patients, and three studies found a decreased QoL for ICD patients. The question of whether QoL relates to ICD therapy cannot be answered conclusively due to the heterogeneity of the existing studies. Lower QoL was apparent among patients with an ICD who experienced several device discharges. Medical staff should be particularly aware of the signs of both psychological and physical disorders in these patients. Further investigations on QoL in ICD patients are desirable, but ethical reasons restrict the conduct of randomized trials.

## Introduction

Since the first implantation of an implantable cardioverter defibrillator (ICD) in a human being in 1980 (1), multiple clinical trials have assessed its impact on mortality and observed beneficial effects among ICD patients (2–9). Consequently, ICD therapy has become a widespread treatment option for patients who are at risk of sudden cardiac death (10, 11). In addition to its impact on survival, the influence of ICD implantation on patients’ health-related quality of life (QoL) has become increasingly important. Improved QoL in cardiac patients after ICD placement might be explained by the reassurance and protection afforded to these patients by their device. Additionally, the ICD may reduce patients’ health concerns and enable them to return to an autonomous and vital lifestyle (12, 13). However, living with an implanted device anticipating or recalling unpredictable and painful shocks may result in feelings of dependence, psychological distress, or fear. Possible consequences include anxiety, depression, or avoidance behaviors, such as self-imposed limitations on physical activities, employment, or driving (14–16). These reactions and aesthetic aspects may result in a reduced QoL among patients with an ICD.

Because the implantation of an ICD is often a prophylactic therapy, not all individual patients who receive such a device will experience lifesaving shocks and derive a survival benefit from it. This fact and the divergent arguments supporting either a reduced or an increased QoL among patients with an ICD warrant an exploration of patients’ actual perceptions of their QoL following ICD implantation. A variety of cardiac patients exist who are alternately treated with an ICD and could potentially serve as a control group. We represent this variety in our systematic review to provide an extensive overview. The primary objective of our systematic review was to investigate whether the health-related QoL of patients with ICDs differs from that of patients who have received medical treatment and from that of patients who have undergone pacemaker implantation or received no intervention. If they were reported in the studies, we also considered ICD shocks or patients’ age as secondary objectives because they are linked to QoL.

## Methods

### Definition of Search Strategy

The methods used in our systematic review are based on the Cochrane Handbook for Systematic Reviews of Interventions (17). Table 1 includes a complete list of search terms. We searched the MEDLINE (accessed via PubMed), EMBASE (accessed via Ovid), Cochrane, Web of Science, and PsychINFO (accessed by Ebsco Host) databases, including both broad bibliographic databases and subject-specific databases. Details on the searched fields within the databases are included in Table 2. The initial search was not limited by any constraints concerning the article type, the language, the date of publication, or the electronic availability of the abstracts.

**Table 1:** **Search terms in the databases**.

(implantable OR internal)
AND
(cardioverter OR defibrillator OR ICD)
AND
(quality of life OR QOL OR adaptation OR acceptance OR attitude to health OR health status OR health state OR psychological OR psychologic OR emotional OR mental OR mood disorder OR mental disorder OR psychiatric disorder OR anxiety OR depression OR depressive OR panic OR fear OR worry OR anger OR frustration OR sadness OR self-doubt OR distress OR stress OR lability OR uncertainty OR concern OR helplessness OR dependence OR hypervigilance OR welfare OR well-being OR wellbeing OR protective OR comfort OR relief OR safety OR independence OR physical OR mobility OR pain OR vitality)

**Table 2:** **The databases and the field search settings used**.

Database	Field search
MEDLINE (accessed by PubMed)	Title/abstract, MeSH terms
Cochrane	Title, abstract, keywords
	Expander: word variations have been searched
EMBASE (Embase Classic + Embase) (accessed by OvidSP)	Title, abstract, subject heading
Web of Science	Title, abstract, author keyword, keywords plus^®^
PsychINFO (accessed by Ebsco Host)	All fields
	Expander: apply related words

### Study Selection

From the initial search results, we included studies in the review if they evaluated the influence of an ICD on QoL, were original studies, and were published in either German or English after the year 2000. Since 2000, all ICD devices have incorporated high-grade technology that is intended to reliably discriminate between supraventricular and ventricular rhythms (1). To be included, patients’ QoL or a closely related endpoint had to be the primary outcome of the study in question. Studies were incorporated irrespective of how QoL was measured and whether they assessed primary or secondary device implantation.

Case studies, cost-effectiveness analyses, systematic reviews, comments, letters, and overview texts were excluded, as were studies without a clearly defined control group without ICD. To provide a broad overview, we did not further specify the type of comparison, though the comparison between single studies became more difficult as a consequence. Possible therapeutic alternatives included no intervention, pacemaker implantation, and medical treatment. We also did not include studies in the review that considered the effects of shocks or device recalls on QoL as their primary objective or studies on resynchronization therapy.

### Selection Procedure

Searches were conducted in all databases on January 13, 2014. Identified records were exported to EndNote X7 and filtered automatically for duplicates. The selection of relevant articles was supervised by a clinical cardiologist. The first level of selection included an independent screening of titles by two reviewers, a health economist and a mathematician. The reviewers’ relative distance from cardiology supported the neutral selection of articles via the application of the defined inclusion and exclusion criteria. The titles of the identified articles were jointly screened, and a decision regarding each article’s inclusion was reached following discussion and the achievement of consensus. The second step of screening entailed the consideration of the abstracts of the remaining articles in a manner similar to that of the first level of selection. The third step entailed full-text screening. The references of the resulting studies, as well as those of each of the systematic reviews identified via the abstract screening, were subsequently hand-searched by one reviewer for further relevant studies.

### Quality Assessment and the Grouping of Studies Using Different Subcategories of QoL

One reviewer assessed the level of evidence of the studies that were included using the Oxford Centre for Evidence-Based Medicine Levels of Evidence (18). Additionally, the study quality was evaluated by the consideration of the risk for bias and confounding and for the existence of a comprehensive and complete report of methodological details and results (++/+/− = high/good/poor methodological quality).

For the aggregation of studies, one reviewer defined different subcategories of QoL (e.g., physical and mental health) based on each of the applied questionnaires and their subscales. For each study, a thorough inspection of the questions contained in the respective questionnaires was conducted to determine which of the defined subcategories would be examined by a particular study.

## Results

**Figure 1:**
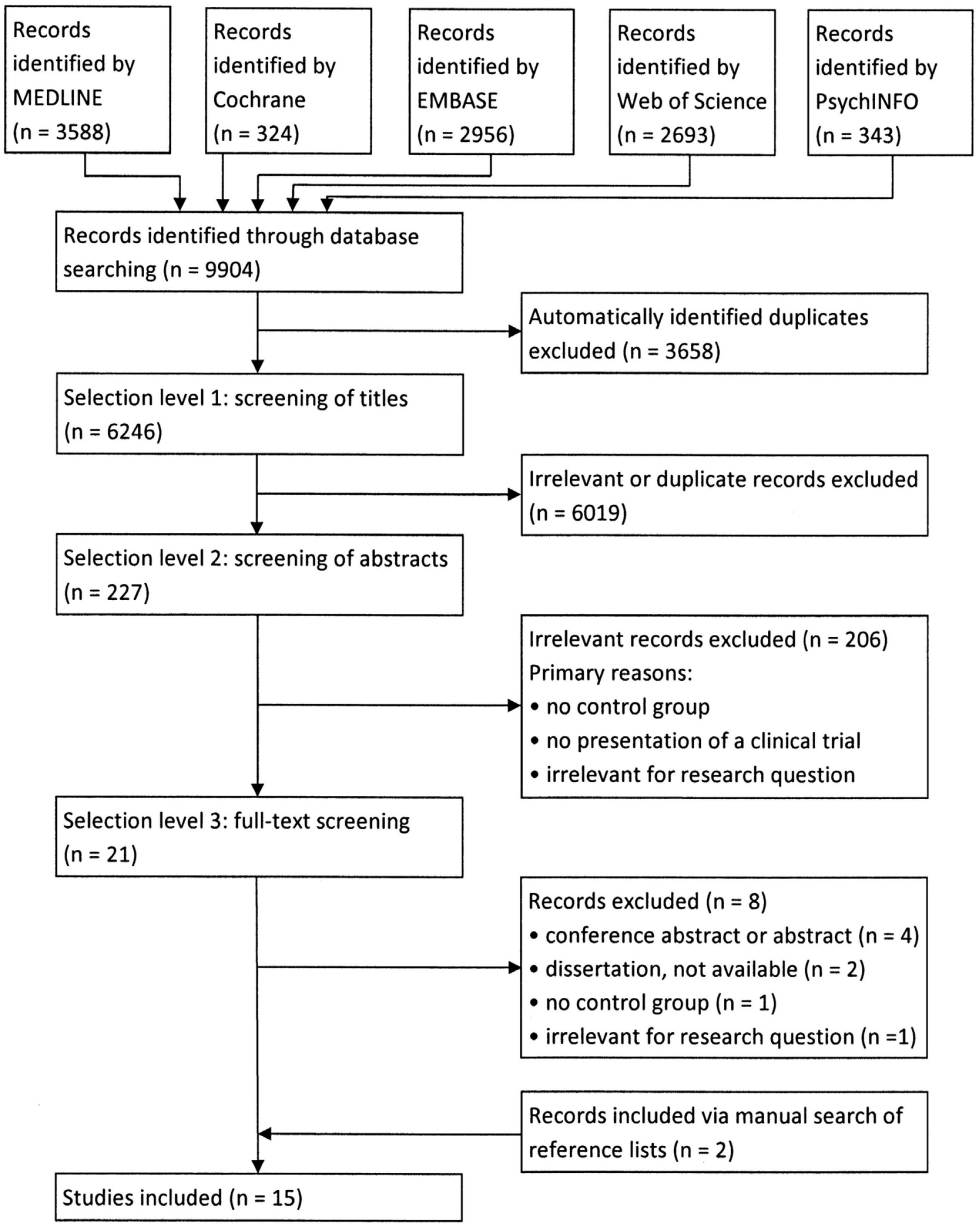
**Literature search flowchart**.

The selection process for the studies included in this review is illustrated in Figure 1. The database search yielded 9,904 records, of which we finally included 15 studies as follows: 5 randomized controlled trials (RCTs) (19–23), 3 observational studies (24–26), and 7 cross-sectional studies (27–33). An overview of study characteristics, findings, and quality assessment is included in Table 3.

**Table 3:** **Characteristics and primary results from the included studies**.

Study, year, country, type of study	Type of prevention: primary/secondary, inclusion and exclusion criteria^a^	Control^b^	No. of patients in study (ICD group, control group), enrollment (E), follow-up (FU)	Conclusion: ICD patients’ QoL compared to control group			LOE/study quality^c^
				Worse	Neutral	Better	
Mark et al. (20) 2008 USA, Canada, New Zealand Multicenter RCT (SCD-HeFT)	Prevention: prim. Inclusion: NYHA class II or III, chronic, stable congestive heart failure due to either ischemic or non-ischemic causes, LVEF ≤ 35% Exclusion: n.r.	Med. (830 patients: conventional medical therapy plus amiodarone, and 833 patients: conventional medical therapy plus amiodarone placebo)	2479 (816, 1663) E: 1997–2001 FU: 30 months		✓
				“In a large primary prevention population with moderately symptomatic heart failure, single lead ICD therapy was not associated with any detectable adverse quality-of-life effects over 30 months of follow-up”			2b++
Noyes et al. (21) 2007 USA Multicenter RCT (MADIT II)	Prevention: prim. Inclusion: prior MI, LVEF ≤ 30% Exclusion: Europeans, missing baseline QoL data, patients from study centers with poor data quality	Med. (conventional medical treatment)	1089 (658, 431) E: 1997–2001 FU: maximum 48 months		✓
				“strong evidence […] [that] the ICD provides little or no quality of life benefits”			2b++
Passman et al. (22) 2007 USA Multicenter RCT (DEFINITE)	Prevention: prim. Inclusion: LVEF ≤ 35% (not caused by CAD), history of symptomatic heart failure, either non-sustained VT or ≥10 premature ventricular depolarizations per hour at baseline Exclusion: n.r.	Med. (conventional medical therapy)	453 (227, 226) E: 1998–2002 FU: maximum 63 months		✓
				“HRQL (health-related QoL) was not affected by ICD implantation in patients in the defibrillators in Non-ischemic Cardiomyopathy Treatment Evaluation study”			2b++
Irvine et al. (19) 2002 Canada Multicenter RCT (CIDS)	Prevention: sec. Inclusion: documented sustained ventricular arrhythmias Exclusion: recent or acute MI or electrode imbalance, not able to read English	Med. (amiodarone)	317 (157, 160) E: 1990–1997 FU: 12 months			✓
				“Quality of life is better with ICD therapy than with amiodarone therapy”			2b+
Hsu et al. (26) 2002 USA Cohort study	Prevention: sec. Inclusion: discharged alive after hospitalization for a live-threatening ventricular arrhythmia, primary diagnosis of cardiac arrest or VT or VF Exclusion: no evidence of cardiac arrest, sustained VT or VF in clinical chart, arrhythmia occurred during the first 48 h after acute MI, non-sustained VT as the only arrhythmia, transient and reversible cause of arrhythmia (drug toxicity, hypoxia, electrolyte imbalance), <18 years, non-English speaking, severe or moderate dementia, life expectancy <6 months, AIDS, discharged to somewhere else but home, no member of Kaiser Northern California System	Med. (91 patients: amiodarone, and 79 patients: antiarrhythmic medications other than amiodarone)	264 (94, 179) E: 1995–1998 FU: 24 months			✓
				“QOL improves more after ICD than after amiodarone therapy”			2b+
Schron et al. (23) 2002 USA Multicenter RCT (AVID)	Prevention: sec. Inclusion: VF or symptomatic VT (including sustained VT resulting in syncope or sustained VT in the setting of LVEF ≤ 40% and clinically important symptoms of hemodynamic compromise), survival of at least 1 year Exclusion: n.r.	Med. (antiarrhythmic drugs)	800 (416, 384) E: n.r. FU: 12 months		✓
				“ICD and AAD (antiarrhythmic drugs) therapy are associated with similar alterations in self-perceived QoL over 1-year follow-up”			2b−
Leosdottir et al. (28) 2006 Iceland Cross-sectional study	Prevention: n.r. Inclusion: all ICD patients living in Iceland at the beginning of 2002 Exclusion: not able to complete questionnaires due to mental or physical disabilities (assessed by caring physician)	PM	108 (41, 67) E: 2002–2003 FU: n.a.		✓
				“ICD patients had a comparable QoL with pacemaker recipients and were not more likely to suffer from anxiety, depression, or general psychiatric distress”			4+
Newall et al. (29) 2007 New Zealand Cross-sectional study	Prevention: n.r. Inclusion: ≥18 years, able to comprehend English Exclusion: taking antidepressant medications for pre-existing depression	PM	95 (46, 49) E: 2005 FU: n.a.		✓
				“Quality-of-life scores were normal for all ICD patients with respect to both mental and physical component scores, and not different from the pacemaker group”			4+
Czosek et al. (31) 2012 USA Multicenter cross-sectional study	Prevention: prim. and sec. Inclusion: children between 8 and 18 and their parents Exclusion: n.r.	PM	173 (40, 133) E: 2004–2008 FU: n.a.	✓
				“Patient- and parent-proxy-reported QOL is significantly affected by the presence of cardiac rhythm devices and is worsened in those patients with CHD (congenital heart disease) and ICD systems as opposed to pacing systems”			4−
Duru et al. (27) 2001 Switzerland Cross-sectional study	Prevention: n.r. Inclusion: 40–70 years, first pectoral implantation of ICD or PM Exclusion: n.r.	PM	152 (76, 76) E: 1993–1999 FU: n.a.		✓
				“There was no difference between the three groups (ICD with experienced shock, ICD without experienced shock, PM), with respect to scores on any aspect of the HAD and SF-36”			4−
Redhead et al. (30) 2010 UK Cross-sectional study	Prevention: sec. Inclusion: first ICD implanted between April 2004 and March 2007 after MI Exclusion: n.r.	PM & Oth. (49 patients: PM, 50 patients: angioplasty, and 50 patients: catheter ablation for drug-resistant atrial fibrillation)	249 (100, 149) E: 2010 FU: n.a.		✓
				“Mean scores for each assessment were similar for each group”			4+
Kamphuis et al. (25) 2002 The Netherlands Observational study	Prevention: n.r. Inclusion: survived an out-of-hospital cardiac arrest due to VT, ≥16 years, able to comprehend Dutch Exclusion: n.r.	Oth. (antiarrhythmic drugs, angioplasty, or surgical revascularization)	168 (133, 35) E: n.r. FU: 12 months	✓
				“In general, OT (other treatment) patients achieved a better quality of life than ICD patients”			2b−
Probst et al. (32) 2011 France Cross-sectional study	Prevention: n.r. Inclusion: Brugada Syndrome (Type 1 ECG before or after a sodium channel blocker challenge) Exclusion: <18 years, no valid mailing address	Oth. (asymptomatic patients without an ICD)	190 (138, 52) E: n.r. FU: n.a.		✓
				“BrS (Brugada Syndrome) patients have a good quality of life with no difference between implanted and non-implanted patients”			4−
Opic et al. (33) 2012 The Netherlands, Belgium Multicenter cross-sectional study	Prevention: n.r. Inclusion: young adults with ToF Exclusion: n.r.	Oth. (ToF patients without an ICD)	54 (26, 28) E: n.r. FU: n.a.	✓
				“ToF patients with an ICD show less favorable psychosocial functioning compared to ToF patients without ICD”			4−
Cross et al. (24) 2010 USA Observational study	Prevention: n.r. Inclusion: ICD therapy and/or diagnosis of CAD Exclusion: obstructive sleep apnea, restless legs syndrome	Oth. (patients with CAD)	60 (30, 30) E: n.r. FU: 14 days			✓
				“The purpose of this study was to compare sleep patterns between CAD and ICD patients […]. The primary and surprising finding was that CAD patients had poorer sleep compared with ICD patients in terms of sleep efficiency and total sleep time”			4−

*^a^n.r., not reported; n.a., not applicable; NYHA, New York Heart Association Functional Classification; LVEF, left ventricular ejection fraction; MI, myocardial infarction; CAD, coronary artery disease; VT, ventricular tachycardia; VF, ventricular fibrillation; ToF, Tetralogy of Fallot*.

*^b^Med., medical treatment; PM, pacemaker implantation; Oth., others or treatment not specified*.

*^c^LOE, level of evidence; 2b, low-level RCT (e.g., no confidence intervals) or individual cohort study; 4, case-series and cross-sectional studies; ++/+/−, high/good/poor methodological quality*.

The studies included in our review varied with respect to the control groups and inclusion and exclusion criteria, as well as the demographic characteristics of the study. For example, one study investigated QoL in children (31), and another study investigated QoL in patients with Tetralogy of Fallot (ToF), with a mean age of 44 years (33). The mean age of the patients who received an ICD in the other studies ranged between 50 and 69 years of age.

The quality assessment revealed frequent methodological limitations, such as an insufficient description of the statistical analyses, the recruitment process, and the existence of missing data and their handling. Furthermore, the problem of multiple testing and confounding variables was often not incorporated into the statistical analyses.

### QoL of ICD Patients Compared with that of Distinct Control Groups

All five of the identified RCTs (19–23) and one cohort study (26) compared patients who underwent ICD implantation with patients who received medical treatment. Medical treatment ranged from conventional medical therapy to treatment with amiodarone. Each of the RCTs assessed all-cause mortality as the primary endpoint and QoL as a secondary endpoint. The number of study participants and the quality of study was intermediate to high. Two RCTs reported improved QoL for patients with an ICD compared to patients with medical treatment. The remaining four studies noted no QoL differences among the groups.

Pacemaker patients served as control subjects in five cross-sectional studies (27–31). Due to various methodological limitations, these studies were assessed to be of moderate to low quality. All studies except the one by Czosek et al. (31) yielded no differences regarding QoL between pacemaker patients and patients with an ICD. Czosek et al. considered pediatric patients and revealed a lower QoL for children with an ICD than for children implanted with a pacemaker.

Redhead et al. (30) compared ICD patients with three groups of patients undergoing other typical cardiac procedures: pacemaker patients, patients who underwent angioplasty, and patients treated via catheter ablation for drug-resistant atrial fibrillation. The remaining four studies (24, 25, 32, 33) selected control subjects from patients receiving antiarrhythmic drugs, patients who underwent angioplasty or patients who underwent surgical revascularization, or had control groups that received an unspecified intervention. Two of these studies found decreased QoL for ICD patients, whereas the other two revealed comparable QoL in ICD and control groups. However, these studies had major methodological limitations. The heterogeneous results must therefore be considered with caution.

### Different Subcategories of the ICD Patients’ QoL

The studies included in our review were arranged according to characteristics that contribute to QoL. These include physical and mental health, e.g., physical limitations, bodily pain, social functioning, anxiety, and depression.

Table 4 shows different subcategories of QoL and the studies that examined these dimensions. Studies were categorized depending on whether improvement or impairment was observed for ICD patients or whether no significant difference was observed. The majority of studies did not show a difference in QoL between ICD patients and controls.

**Table 4:** **Findings from the included studies with respect to distinct subcategories of QoL**.

Subcategory of QoL	Mark (20)	Noyes (21)	Passman (22)	Irvine (19)	Hsu (26)	Schron (23)	Leosdottir (28)	Newall (29)	Czosek (31)	Duru (27)	Redhead (30)	Kamphuis (25)	Probst (32)	Opic (33)	Cross (24)
**Physical health (physical limitations, bodily pain, and self-perception of general health status)**			n.s.			n.s.		n.s.		n.s.					
Physical functioning (focus: physical limitations)	n.s.	n.s.		+	n.s.				n.s.	n.s.		n.s.	n.s.	−	n.s.
Physical role (focus: problems with work or other activities due to physical limitations)	+									n.s.		n.s.	n.s.	n.s.	
Bodily pain	+			n.s.						n.s.		−	n.s.	n.s.	
Sleep efficiency and quality				+											+
Self-perception of general health	+									n.s.		n.s.	n.s.	−	

**Mental health (social and emotional functioning, psychological well-being, vitality)**	+		n.s.	+		n.s.	n.s.	n.s.	n.s.	n.s.					n.s.
Social functioning (focus: impact of physical or emotional problems on social activities)	+			n.s.						n.s.		n.s.	n.s.	n.s.	
Emotional role (focus: limitations at work or other activities due to emotional problems)	+									n.s.	n.s.	n.s.	n.s.	n.s.	
Psychological well-being (focus: anxiety, depression, mental health)							n.s.			n.s.	n.s.	n.s.	n.s.	n.s.	
Vitality (focus: subjective well-being, e.g., energy level, fatigue)	n.s.			+	n.s.					n.s.		n.s.	n.s.	n.s.	
Satisfaction with life														−	
**Anxiety**							n.s.	n.s.		n.s.	n.s.	n.s.	n.s.	n.s.	n.s.
**Depression**				+			n.s.	n.s.		n.s.	n.s.	n.s.		n.s.	

Reassurance by ICD								n.s.		+					
Perception of being informed about ICD								n.s.		n.s.			n.s.		
Bodily awareness														n.s.	

*+, better for ICD group; −, worse for ICD group; n.s., not significant; blank fields, not investigated or not reported*.

### Relationship Between QoL and Age in ICD Patients

The cross-sectional study by Czosek et al. (31) and the RCT by Passman et al. (22) found no correlation between QoL and patients’ ages. Opic et al. (33) reported worse psychosocial functioning in younger patients. Poor QoL was noted among elderly patients by Hsu et al. (26). Kamphuis et al. (25) reported improved vitality but poorer health perception among elderly ICD patients. Due to the heterogeneity of these conclusions, it was not possible to elucidate any trends regarding the relationship between age and QoL in patients with an ICD or to determine whether this relationship differed among ICD recipients compared with the general population.

### Relationship Between QoL and Shocks in ICD Patients

All but two studies investigated whether a correlation exists between the perceived QoL of patients with an ICD and delivered shocks. Four cross-sectional studies and one cohort study did not observe any differences in health-related QoL between patients with an ICD who had or had not experienced a shock (26–29, 31). In the SCD-HeFT trial, Mark et al. (20) also did not observe a difference in QoL, with the exception of those patients who were shocked within a month of their QoL assessment. This finding suggests that the negative influence of ICD shocks on QoL decreases with time. Due to the small number of patients whose QoL was measured within a month of ICD shock, this finding remains to be reproduced.

Two RCTs reported that QoL of ICD patients depended on the number of shocks received. Irvine et al. (19) and Passman et al. (22) found a reduced mental well-being only for patients who experienced five or more shocks.

Three cross-sectional studies (30, 32, 33) revealed a poorer psychological well-being for ICD patients following shocks, including psychosocial problems, anxiety, and concerns regarding complications. Kamphuis et al. (25) demonstrated decreased physical functioning among patients who had received a shock. Schron et al. (23) observed lower physical and mental scores among the patients of the AVID trial with a history of ICD discharge.

## Discussion

In general, we could not ascertain a uniform trend regarding the QoL of patients who received an ICD. The majority of studies concluded that there was no difference between the ICD groups and the control groups. For the subgroup of patients who experience ICD shocks, the data suggest unchanged or poorer QoL compared to ICD patients without ICD discharge. A high number of shocks and the recency of shocks appear to correlate with reduced QoL. However, an underlying causality cannot be deduced from this correlation. It is possible that repeated shock experiences will influence a patient’s perception of his health in a negative way. It is also possible that sicker patients with poorer QoL are increasingly affected by shocks.

Due to differences in control groups, study designs, and inclusion and exclusion criteria, there was limited comparability and heterogeneous methodological quality among the studies reviewed. The three studies that were assessed to be of high quality and to have a high level of evidence were the SCD-HeFT (20), the MADIT II (21), and the DEFINITE (22) studies, which included a total of 4,021 patients. These three RCTs found similar QoL between ICD patients and controls under medical treatment. Comparable results were also published in further studies, but the reader should keep in mind the frequent differences especially in the underlying patient structures. For example, Probst et al. (32) detected no relevant differences of Qol in ICD patients and controls as well, but compared to SCD-HeFT (20), MADIT II (21), and DEFINITE (22), this investigation is focused on patients suffering from Brugada syndrome. These patients had a relevant lower age (50–54 years) compared with the mean age of ICD patients in SCD-HeFT, MADIT, and DEFINITE (ranging from 59 to 64 years). Additionally, Brugada patients are less suffering from comorbidities and are more frequently professionally active (32).

Because several RCTs have demonstrated that ICDs exert a survival advantage over medical treatment (2–9), the allocation of ICD therapy and medical treatment via randomization is ethically questionable. Therefore, the RCTs identified by our systematic review were conducted over 10 years ago. The more current studies included in our review were observational studies and were characterized by a lower evidence level. Their small patient numbers and their single-center and cross-sectional designs further diminished the informative value of these studies. The longitudinal studies included in our review had short follow-up (FU) periods. Three of the six longitudinal studies followed patients for only 12 months (19, 23, 25). The FU periods of the remaining studies lasted as long as 30, 48, or 63 months (20–22). Because QoL is particularly influenced by incisive experiences, including device implantation, and habituation, a FU period of several years may be more appropriate.

Two additional limitations of the studies that assessed the relationship between ICD implantation and various outcomes are the impossibility of blinding and the definition of a suitable control group. Unblinded cardiologists do not ensure the equal treatment of patients who are receiving distinct therapies. Hence, there exists a high risk of bias. This is particularly important when assessing an outcome such as QoL, which is often influenced by a patient’s contact with medical staff. The selection of a control group for an ICD population often requires relatively large compromises. Patients with an implanted pacemaker, for instance, often differ in both their demographic and clinical characteristics. They are usually older than patients with an ICD and are more likely to be women.

As a procedural limitation of the development of our review, the screening of titles as an initial selection step must be mentioned. This step is more prone to missing relevant articles than screening of abstracts. An additional issue is the inclusion of newer studies that were published only following the year 2000. In some cases, there existed a time lag of several years between the completion of the study and the publication of the results. Hence, there were studies that investigated patients much earlier than the publication date (19, 27). Consequently, the technology of the implanted devices and the patients’ awareness and acceptance of ICD therapy may not have been comparable. Finally, our review did not include systematic reviews and meta-analyses, which were, however, used later to check the thoroughness of our review.

Previous reviews and meta-analyses on QoL in ICD patients drew opposing conclusions. McCready et al. (34) concluded that ICD implantation was superior to medical therapy with respect to QoL, based on eight studies published between 1995 and 2002. Groeneveld et al. (16), who included 27 studies published between 1995 and 2005, came to the same conclusion. Additionally, they emphasized that changes in QoL were strongly dependent on the comparison groups. Compared with the general public or pacemaker patients, ICD recipients had a lower QoL. Unchanged or improved QoL was noted for ICD patients based on pre- and post-implant comparisons by Shea (15), who reviewed three trials conducted in the 1990s. Francis et al. (35) included 30 studies that were published between 1993 and 2004. Five randomized trials suggested either an unchanged or an improved QoL among the patients who underwent an ICD implantation. The 16 non-randomized trials under study showed a balanced result. In their meta-analysis, Burke et al. (36) concluded either unchanged or poorer QoL for patients who received an ICD. They considered 20 publications, each of which was published before the year 2000, using various comparisons, including pre- and post-implantation comparisons and comparisons with cardiac patients who did not receive therapy and patients who received antiarrhythmic drug therapy.

Our systematic review confirmed the trend observed by McCready et al. (34) and Groeneveld et al. (16), as we determined an either unchanged or improved QoL for ICD patients compared with patients under optimal medical treatment; however, either unchanged or poorer QoL was noted among ICD patients compared with pacemaker recipients. However, due to both the small number of studies and various limitations concerning both methodology and implementation, we do not view this trend as firm but rather as a finding that remains to be reproduced.

The value of pre- and post-implantation QoL comparisons appears to be limited. We expect an imminent implantation to strongly influence patients’ QoL and their psychological well-being in particular. QoL may decrease due to worries about the surgical operation or increase due to the expected improvement of state of health owing to the ICD. Therefore, we focused on the comparison between patients who received an ICD and a non-ICD control group in our systematic review.

The heterogeneous results of the studies available for our review did not permit a definitive answer to the question of whether health-related QoL differs between patients with an ICD and respective controls. One may remain critical of whether patients with pacemakers or patients who have received medical treatment are representative as control patients. Lower QoL was apparent among ICD patients who experienced several device discharges. Medical staff should be particularly aware of psychological and physical effects in these patients. Future research on these open QoL questions is difficult because it is currently not ethical to randomize patients to ICD or control treatment.

## Author Contributions

JT contributed to concept and design, data collection, screening of literature, summarizing and interpreting literature, drafting article, critical revision of the article, and approval of the article. KK contributed to concept and design, critical revision of the article, and approval of the article. MZ contributed to supervision of screening of literature, critical revision of the article, and approval of the article. SW contributed to critical revision of the article and approval of the article. TR contributed to concept and design, data collection, screening of literature, summarizing and interpreting literature, critical revision of the article, and approval of the article.

## Conflict of Interest Statement

Juliane Tomzik: none. Katharina C. Koltermann: consultancies as part-time manager for Boston Healthcare Associates, external scientific consulting services for Takeda, CGS Clinical Guideline Services, Keimzelle Medical Ventures, and Scenarium Group GmbH. Markus Zabel: consultancies, research and travel grants: Biotronik, Medtronic, Boston Scientific. Stefan N. Willich: none. Thomas Reinhold: received fees from ICD manufacturer Biotronik for external scientific consulting services.
